# Systems Biology in the study of Neurological Disorders: Focus on Alzheimer’s Disease

**Published:** 2008

**Authors:** Giulio M. Pasinetti, Susanne Hiller-Sturmhöfel

**Keywords:** Alzheimer’s disease (AD), cognitive impairment, dementia, genetic risk factors, biomarkers, complementary DNA (cDNA) microarray analysis, protein array analysis, systems biology, proteomics, alcohol and other drug effects (AODEs)

## Abstract

Systems biology approaches may be useful for studying the mechanisms underlying alcohol’s harmful effects on the brain. Such approaches already are used in the study of Alzheimer’s disease (AD), a progressive neurodegenerative disorder that, with the overall increase in life expectancy, will affect an increasing proportion of the population and become an increasingly serious public health concern. Systems biology approaches such as complementary DNA (cDNA) microarray analyses have helped identify several genes whose expression is altered in patients exhibiting the earliest stages of AD. Several of these genes are involved in the release of messenger molecules from the ends of nerve cells (i.e., in synaptic vesicle functioning), and their particular role in AD must be investigated further using conventional molecular biological approaches. Similarly, protein array analyses have identified candidate proteins that may play a role in the development of AD. Finally, proteomic approaches, such as certain mass spectrometry techniques, have been used to search for biomarkers of the progression from normal cognitive functioning to mild cognitive impairment and AD, which eventually may allow early and reliable diagnosis of the disease. These approaches already have yielded some candidate molecules whose validity and reliability as biomarkers of AD, however, still need to be confirmed.

As the articles in this issue of *Alcohol Research & Health* demonstrate, systems biology approaches increasingly are being used in research on several disorders associated with excessive alcohol use. One of the organs most severely affected by excessive alcohol consumption is the brain, and long-term heavy drinking has serious detrimental effects on brain structure and functioning. For example, alcoholics often exhibit various degrees of cognitive impairment and, in the most severe cases, can develop alcoholic dementia. In addition, some alcoholics develop a condition known as Wernicke-Korsakoff syndrome, which is characterized by shrinkage of brain tissue and memory loss (i.e., anterograde amnesia). Systems biology approaches may be useful for studying the mechanisms underlying alcohol’s harmful effects on the brain as well as its consequences and ultimately aid in the diagnosis of alcohol-related neurological deficits. Although these approaches only are beginning to be used in research on alcohol-related neurological disorders, they already have been employed in the study of other neurological disorders, most prominently Alzheimer’s disease (AD), for which genomic and, particularly, proteomic approaches may help identify biomarkers that allow physicians to reliably diagnose the disease at an early stage. Accordingly, AD can be used as an example to illustrate the potential of similar approaches for the understanding and perhaps diagnosis of alcohol-related neurological deficits. Furthermore, this research on AD may be relevant to alcohol research because some investigators have suggested that alcohol use may influence the risk of developing AD, as certain brain-signaling systems are affected both by alcohol use and by AD (Tyas 2001). To date, however, epidemiological studies have not found strong evidence to support an association between alcohol use and AD (Tyas 2001).

After providing a brief background on AD, this article describes the systems biology approaches that have been used in the study of AD and summarizes the most relevant findings and perspectives. Although this discussion focuses only on one particular disorder and on the work of only a few research groups, it highlights the potential of systems biology approaches for improving diagnosis and understanding of neurological disorders in general, including those associated with alcoholism.

## What is AD?

AD is a progressive neurodegenerative disorder characterized by a gradual destruction of brain cells that results in a progressive decline in mental functions (e.g., memory or ability to learn, reason, make judgments, and communicate), culminating in severe dementia. At advanced stages, this decline leaves the patient unable to carry out daily activities, such as dressing, eating, or personal hygiene. Moreover, the patient’s personality and behavior may change so that he or she becomes more anxious, agitated, suspicious, or even aggressive.

In addition to these mental and behavioral manifestations, AD is associated with characteristic physiological changes in the patient’s brain that may begin to develop years before the first symptoms of impaired mental function become noticeable. By the time the mental and behavioral symptoms appear, irreversible damage to and death of nerve cells in certain brain areas already may have occurred, with the damage progressively spreading through different brain regions. In addition to these gross anatomical changes, two characteristic structural abnormalities are found in the brains of AD patients:

- *Amyloid plaques*—clumps of protein fragments (Aβ-peptide) that are derived from a protein called amyloid precursor protein (APP), which is embedded in the membranes surrounding the nerve cells; the Aβ clumps accumulate outside the brain cells;
- *Neurofibrillary tangles*—twisted strands of a protein called “tau” that accumulate within the brain cells.

It is not clear, however, if the formation of these structural abnormalities precedes, coincides with, or follows the earliest, mildest (and often unrecognized) signs of declining mental function.

### Causes of AD

There is no single known cause of AD. In the vast majority of cases, AD affects people older than age 65. Accordingly, age is the greatest known risk factor for developing AD (Alzheimer’s Association 2006). This also implies that with the overall aging of the population, particularly of the baby boomer generation, AD will become an increasingly important public health concern.

A family history of AD also increases a person’s risk of developing the disease, suggesting that genetic and/or environmental factors play a role. Researchers are trying to identify these genetic and environmental factors. These studies have led to the identification of one gene called apolipoprotein E-e4 (APOE-e4) that increases a person’s risk of developing AD (Kukull et al. 1996). However, not all people who carry this gene develop AD, and several other genes likely also are involved. Moreover, it still is unknown exactly how the APOE-e4 gene increases AD risk. Therefore, researchers still are searching for additional genes that may increase AD risk as well as for the mechanisms of action of these genes.

A few cases of AD are caused by specific rare genes, and people who inherit these genes are virtually certain to develop AD, often as early as their 30s and 40s (Alzheimer’s Association 2006).

### Diagnosis of AD

Although the presence of amyloid plaques and neurofibrillary tangles is considered the most specific indicator of AD, it cannot be used in the diagnosis of AD patients because these structural abnormalities only can be seen at autopsy and cannot be detected through current brain-imaging techniques. Instead, physicians must rely on symptoms reported by the patient or family members, such as severe forgetfulness, confusion, or getting lost in familiar places. Based on this information, as well as the patient’s medical history, physical exams, and assessments of neurological and mental status, physicians in most cases can make a diagnosis of probable or possible AD. Nevertheless, many AD patients remain undiagnosed or are diagnosed only late in disease progression. Early diagnosis, however, is important because medications and other treatment modalities are available that, although not offering a cure, may slow the progression of the disease and the patient’s mental decline. Moreover, the current diagnostic process is time-consuming and costly. Accordingly, developing additional diagnostic approaches that do not rely on self- or family reports or behavioral observation, such as the identification of easily assessable biomarkers, remains a high priority of AD research.

To address this and other questions regarding risk factors for the development and progression of AD, researchers increasingly are using systems biology approaches that generate and combine data through a variety of experimental strategies at different levels of investigation (e.g., at the molecular, cellular, or tissue level). As described in the following sections, these include complementary DNA (cDNA) microarray analyses, as well as proteomic strategies, to identify the genes and proteins involved in these processes.

## cDNA Microarrays Help Identify Genes Involved in AD

One important area of AD research focuses on identifying genes that increase a person’s risk of developing AD or that may play a role in the onset or progression of AD. To this end, researchers at the Mount Sinai School of Medicine in New York are studying cDNA expression patterns in patients with known mental status—no dementia, questionable dementia, mild dementia, or moderate dementia (which corresponds to early or mild AD)—for whom brain samples are available after death.

Not all of a person’s genes are active (i.e., expressed) all the time. At any given time and in any given tissue or cell, only a subset of genes are expressed. This means that the genetic information encoded in those genes is copied into an intermediary molecule called messenger RNA (mRNA) in a process known as transcription. The mRNA then is used as a template to produce the protein products encoded by the genes. Researchers can isolate the mRNA generated in a cell type or tissue and copy it into molecules known as cDNA, which then can be used for further analysis. Thus, the cDNA generated from a certain tissue type (e.g., brain tissue from certain brain regions obtained at autopsy) represents the genes that were active in that brain region. By comparing cDNA patterns in the brains of people with a disease (e.g., dementia) with those of people without the disease (e.g., normal mental function), researchers can identify genes whose expression varies between the two groups and which, therefore, may be related to the disease.

Ho and colleagues (2001) employed this approach to identify genes whose expression may be altered in patients with moderate dementia, using high-throughput cDNA microarrays to screen almost 6,800 genes. The researchers identified 32 cDNAs whose levels differed by at least 1.8-fold between patients with normal cognitive function and those with moderate dementia (i.e., early AD). Of these 32 cDNAs, 25 belonged to known genes and 7 were DNA fragments that are obtained during cDNA generation but whose biologic function is unknown. In most cases, cDNA levels (i.e., expression of the corresponding genes) were lower than normal (i.e., downregulated) in dementia patients compared with neurological control patients.

Interestingly, one of the known genes whose expression was downregulated encodes a molecule called synapsin II, which normally plays an important role in nerve signal transmission (Ho et al. 2001). Signal transmission between nerve cells (i.e., neurons) occurs at a specialized region of the signal-emitting neuron called the synapse. The synapse contains small bubbles (i.e., vesicles) in which signaling molecules (i.e., neurotransmitter molecules) are stored. When a nerve signal arrives at the synapse to be transmitted to a neighboring neuron, the vesicles move to the membrane surrounding the neuron and release their neurotransmitter contents into the space between the signal-emitting and signal-receiving neuron, so that the neurotransmitter molecules can migrate to the signal-receiving neuron and initiate their specific effects there. The empty vesicles then must be recycled and refilled with neurotransmitter molecules to be available for transmitting subsequent nerve signals. Synapsin II, which was found to be downregulated in dementia patients, is involved in synaptic vesicle metabolism and neurotransmitter release. Accordingly, down-regulation of synapsin II expression could interfere with normal nerve signal transmission and may contribute to the dementia and other changes in brain function associated with AD.

More detailed analyses demonstrated that synapsin II downregulation does not occur in all brain regions of dementia patients but is limited to those regions that are at greatest risk of developing the damage characteristic of AD. Brain regions that typically are spared from AD damage also exhibited no downregulation of synapsin II expression (Ho et al. 2001). These findings further support the hypothesis that downregulation of synapsin II expression may be one of the mechanisms contributing to the development of dementia and, subsequently, AD.

The potentially central role of synaptic vesicle function in the development of AD has been further supported by the findings of Yao and colleagues (2003), who also conducted cDNA microarray analyses of brain tissue from AD patients. These researchers observed a reduced expression of a group of genes related to vesicle transport to the cell membrane and back into the cell (i.e., synaptic vesicle trafficking). Conversely, the expression of several other genes that are involved in synaptic functions other than vesicle trafficking was not altered in the AD patients compared with control subjects.

Finally, Lorin and colleagues (2001) used cDNA microarray analyses to compare affected and unaffected brain tissue obtained after death from six patients with AD and nine control subjects. Again, these researchers found that genes related to synaptic vesicle synthesis and function were downregulated in the affected AD brain tissue, as were genes related to signal transduction, energy metabolism, stress response, calcium binding, and the molecules that help cells maintain their shape (i.e., the cytoskeleton). At the same time, some genes related to chronic inflammation, adhesion of cells to one another, cell proliferation, and protein synthesis were upregulated.

Thus, cDNA microarray analyses have identified several genes that appear to be important in the early stages of AD and which can be studied further using conventional approaches to determine their exact role(s) in the development and/or progression of AD. The results provide evidence that altered gene expression of selective genes already occurs during the earliest detectable stages of AD dementia. For example, altered regulation of synapsin-mediated neurotransmitter release in selected brain regions may influence the cognitive decline during early AD.

However, cDNA microarray analyses only provide a “snapshot” of gene expression (i.e., of the steady-state mRNA levels) in a given tissue at a specific time and under specific conditions. It is possible, however, that processes occurring after the transcription of DNA into mRNA—that is, during the generation of protein molecules from the mRNA template (i.e., translation) and during further processing of the generated proteins—also differ between people with and without AD and may contribute to AD development and progression. Therefore, cDNA microarray technology approaches alone likely provide only a partial view of the biological processes leading to AD. Consequently, the combination of cDNA and protein array analyses now has become the gold standard for research aimed at a more global understanding of complex biological processes, such as the pathogenesis of AD. The following section discusses some of the findings obtained with proteomic studies of AD.

## Protein Array Studies Identify Candidate Proteins Involved in AD

To identify differences in protein expression between people with mild cognitive impairment (who are at high risk of developing AD) and people with normal cognitive functioning, Ho and colleagues (2005) used commercial protein arrays (i.e., PowerBlot arrays) that allowed for the screening of 750 independent proteins in a single assay. These analyses found 50 candidate proteins whose levels differed consistently and reproducibly between patients with moderate cognitive impairment and normal control subjects. Of these, the investigators were able to identify 23 proteins, most of which fell into one of five groups with respect to their function. These five groups included the following:

- Proteins related to neurotransmitters and synaptic function;
- Proteins related to the cytoskeleton and adhesion of cells to one another;
- Proteins related to the cell cycle (i.e., involved in growth and multiplication of cells as well as in cell death);
- Proteins related to apoptosis, or programmed cell death; and
- Proteins related to transcription and translation.

For most of these proteins, levels were at least two-fold lower in patients with cognitive impairment than in control patients; only for a few proteins were levels higher in the cognitively impaired patients than in the control subjects. These findings are consistent with those obtained in cDNA arrays in that both cDNA and protein array studies point to central roles of genes and proteins involved in neurotransmitter and synaptic function, the cytoskeleton and cell adhesion, and cell proliferation in the development or manifestation of AD.

The researchers then used traditional techniques to evaluate the levels of these proteins in a larger number of samples from patients with and without cognitive impairment. These analyses identified, in particular, a protein called tomosyn, whose levels consistently showed a decrease by at least three-fold in the cognitively impaired patients (Ho et al. 2005). Tomosyn also is important in the metabolism and refilling of the synaptic vesicles, further supporting the hypothesis that reduced synaptic functions may be associated with the cognitive impairment in patients with moderate cognitive impairment. (The synapsin II protein, whose cDNA levels were found to be reduced in the cDNA microarray studies, was not among the 750 proteins studied here; therefore, its protein levels could not be evaluated.) Thus, it appears that cDNA microarray and protein array studies can complement each other in the search for molecular markers of cognitive impairment and early AD.

## Proteomic Approaches Help Identify Biomarkers of AD

One important aspect of current AD research is the search for proteins whose presence or expression levels differ consistently between people with and without cognitive impairment or AD and that easily can be measured so that they can be used by physicians to diagnose these conditions and monitor their progression (i.e., biomarkers of AD). Thus, biomarker discovery studies focus on identifying protein profiles that ultimately can be used to develop rapid, sensitive, and specific high-throughput diagnostic tests. To achieve this goal, such biomarkers ideally should be present in biological fluids (e.g., blood or cerebrospinal fluid [CSF])^1^ and not only in brain tissue because these fluids can be attained much more easily than brain tissue samples. However, the identification of such biomarkers is challenging because they must meet at least two requirements:

- Their levels must be high enough so that they can be detected among all the other proteins found in these biological fluids.
- They must be specific to the condition in the brain being studied; many of the proteins circulating in the blood or CSF can be produced by multiple cell types and different organs and therefore lack this specificity.

In their efforts to identify suitable biomarkers of the progression from normal cognitive functioning to mild cognitive impairment and AD, researchers have begun to use protein chip arrays whose surfaces have been treated chemically or biochemically so that the arrays can bind only proteins with certain structural or functional characteristics. Then, crude protein extracts from the tissue or fluid of interest are applied to the arrays, and the proteins that are bound by the arrays are analyzed using mass spectrometry approaches, such as surface-enhanced laser desorption/ionization time-of-flight (SELDI-TOF). (For a more detailed discussion of mass spectrometry techniques, see the article by Hiller-Sturmhöfel, Sobin, and Mayfield on p. 36.) If samples from patients with the disease and control subjects are run in parallel, differences in protein profiles can be identified using computerized analyses. This strategy allows for highly sensitive, quantitative, and reproducible analysis of protein expression patterns and may offer a novel means for identifying and eventually monitoring the onset and progression of AD and the associated dementia (Ho et al. 2005). Using this approach, investigators are analyzing several potential biomarkers.

One approach has focused on determining the levels of Aβ-peptide in biological fluids. As mentioned earlier, one of the characteristic features of AD is the formation of amyloid plaques in the brain that consist mainly of Aβ-peptide. Several variants of Aβ-peptide exist, and precise determination of the patterns of these various Aβ-peptide species in tissue extracts or body fluids would provide a classical example of a prognostic biomarker and contribute to the understanding of AD pathogenesis. Therefore, in addition to traditional molecular biological techniques, researchers have used the protein chip array/SELDI-TOF technique to simultaneously detect the various Aβ-peptide variants in order to gain a better understanding of the processes involved in AD development and progression. To date, this strategy has been used to study AD pathogenesis (Davies et al. 1999; Goldstein et al. 2003; Vehmas et al. 2001; Xiang et al. 2002) and the processes occurring during the breakdown of APP into the Aβ-peptide species (Austen et al. 2000; Beher et al. 2002; Freares et al. 1999). Other studies using this strategy were aimed at exploring new drugs for AD treatment (Beher et al. 2002; Davies et al. 1999) or potential development of a vaccine therapy for AD (Vehmas et al. 2001).

Other efforts in the search for biomarkers of AD have focused on determining whether a combination of several proteins could serve as a diagnostic biomarker of AD, eventually leading to a test that could easily distinguish patients with cognitive impairment or AD from cognitively normal elderly patients and distinguish between AD and other types of dementia. One group of researchers reported that, using protein chip arrays and SELDI-TOF, they identified a panel of five proteins whose expression was significantly altered in AD patients compared with healthy control subjects (Carette et al. 2003). Other researchers proposed using the ratio of different variants of the Aβ-peptide in the CSF as a diagnostic tool for distinguishing patients with AD from healthy people and patients with other forms of dementia (Shoji 2002); however, this approach appears to be less specific and sensitive than the one using the panel of five proteins.

Finally, researchers are evaluating the potential of protein chip arrays and SELDI-TOF mass spectrometry for identifying novel protein biomarkers that can be used as indicators of the onset and eventual progression of the cognitive decline associated with early AD. Using protein microchips that had been treated so that they selectively interacted with negatively charged or copper-binding proteins, Ho and colleagues (2002) detected a novel protein biomarker—a molecule known as S100–A7 or psoriasin—whose function still is unknown but whose levels were increased in the CSF of patients with early mild cognitive impairment but not in control patients. Additional studies currently are exploring whether the levels of this protein in the CSF are regulated as a function of the progression of dementia, and initial results suggest that psoriasin levels do indeed increase with advancing dementia.

## Future Perspective

System biology approaches such as high-throughput cDNA and protein microarrays have begun to help researchers discover the mechanisms involved in neurobiological diseases such as AD and also schizophrenia (Marcotte et al. 2003). For example, as described here, several studies have sought to identify biomarkers that can be used to diagnose or predict the progression of cognitive impairment and AD. Although these new technical approaches already have yielded some interesting results, it is important to use them with great care and rigor and to validate the findings using traditional approaches in order to avoid identification of false-positive markers that in fact are not associated with the disease under investigation and which could lead to the misdiagnosis of many patients. Despite these reservations, it appears plausible that similar approaches can be used to search for biomarkers of other neurological disorders, including those associated with alcoholism.

cDNA as well as protein array studies also have led to the identification of several genes and proteins (e.g., synapsin and tomosyn) that appear to play a role in the development of cognitive impairment and AD. The validity of these findings is supported by the fact that the results obtained with both approaches point to the same cellular process (e.g., the functions and recycling of synaptic vesicles). Similarly, it might be possible to identify the processes involved in the neurological consequences of alcoholism, particularly because alcohol’s effects on cognition, brain disorders, and brain chemistry appear to share some features with AD’s effects on these three areas (Tyas 2001).

Finally, cDNA and proteomic studies may help elucidate the effects of novel therapeutic agents and other factors that influence the course of AD and other neurological disorders. This aspect of AD research also might offer some interesting connection to the alcohol research field because researchers recently reported that moderate consumption of red wine may have a beneficial role in patients with neurodegenerative disorders such as AD. Using a mouse model of AD, Wang and colleagues (2006) demonstrated that moderate consumption of red wine reduced the deterioration of the animal’s spatial memory as well as the formation of amyloid plaques. However, neither the specific compounds found in the wine that were responsible for this effect nor their mechanism(s) of action have been clearly identified to date. Traditional molecular biological techniques, combined with cDNA and protein microarrays, may help researchers determine both the wine components involved and the genes and/or proteins whose functions are modified by these compounds and contribute to the beneficial effects on AD progression.

All of these examples demonstrate the great potential that systems biological approaches have for enhancing understanding, diagnosis, prognosis, treatment, and prevention not only of AD but of a range of neurological disorders, including alcohol-related conditions. The full realization of this potential has only just begun.

## Footnotes

1 CSF is the fluid that bathes the brain and spinal cord. Although it cannot be sampled as easily as the blood, procedures to obtain it are much less invasive than those for obtaining brain tissue samples.
